# Tailor-made multiple sequence alignments using the PRALINE 2 alignment toolkit

**DOI:** 10.1093/bioinformatics/btz572

**Published:** 2019-08-01

**Authors:** Maurits J J Dijkstra, Atze J van der Ploeg, K Anton Feenstra, Wan J Fokkink, Sanne Abeln, Jaap Heringa

**Affiliations:** Department of Computer Science, Vrije Universiteit, 1081 HV Amsterdam, The Netherlands

## Abstract

**Summary:**

PRALINE 2 is a toolkit for custom multiple sequence alignment workflows. It can be used to incorporate sequence annotations, such as secondary structure or (DNA) motifs, into the alignment scoring, as well as to customize many other aspects of a progressive multiple alignment workflow.

**Availability and implementation:**

PRALINE 2 is implemented in Python and available as open source software on GitHub: https://github.com/ibivu/PRALINE/.

## 1 Introduction

Multiple sequence alignment (MSA) is one of the fundamental tasks in bioinformatics, essential to a wide variety of workflows, including fold prediction, phylogenetic analysis and mutation impact prediction. The exact solution with dynamic programming is not feasible for more than a handful of sequences. For protein and small- to medium-sized nucleotide sequences, MSA is therefore performed by iteratively applying the dynamic programming algorithm on pairs of sequences to grow a multiple alignment, in what is called progressive multiple alignment (Hogeweg and Hesper, 1984).

A multitude of advanced alignment programs exist (Nakamura *et al.*, 2018; Sievers and Higgins, 2018; Simossis and Heringa, 2005), but improvements have mostly been focused on improving the heuristics of progressive alignment (Heringa, 1999; Sievers *et al.*, 2014), and on the accuracy of the scoring in the pairwise alignment step (Alva *et al.*, 2016; Henikoff and Henikoff, 1992). In many cases, however, it is already known that some subregions of a sequence should be aligned, for example on the basis of a conserved functional motif or secondary structure element. A suitable alignment program, when provided with these kinds of annotations to the primary sequence, could use the additional conservation signal to improve alignment quality.

Here we present PRALINE 2, a toolkit for this kind of tailored alignment problem. PRALINE 2 supports arbitrary sequence alphabets, and allows multiple alphabets to be used simultaneously, such as DNA and protein sequences with corresponding sequence motifs. PRALINE 2 is a reimplementation of the PSI-PRALINE program (Simossis and Heringa, 2005) and has out-of-the-box support for many of the commonly used algorithms in multiple sequence alignment. It was written from scratch in well-documented, modern code, and should be easy to extend or adapt for a particular use case.

## 2 Materials and methods

**Fig. 1.**
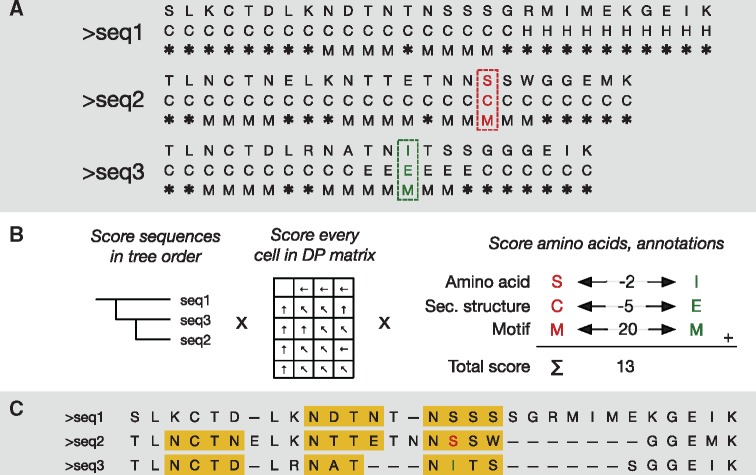
Overview of the PRALINE 2 algorithm, showing how an alignment can be improved by incorporating sequence annotations. (**A**) Three input amino acid sequences alongside two annotation tracks: the 3-state secondary structure (C, E, H) and an annotation predicting whether an N-terminal glycosylation site exists at a position (M) or not (*). A pair of columns, shown in red and green, is tracked throughout the steps of the algorithm. (**B**) Scoring in more detail. The three types of symbols contribute independently to the total score: amino acids are scored by BLOSUM62, secondary structure by a 5/-5 match/mismatch scheme, and, if both positions are a glycosylation motif, a score boost of 20 is applied. (**C**) The resulting alignment (amino acid sequences only); note that the motifs, shown in yellow, are correctly aligned, due to the motif scoring

Central to the architecture of PRALINE 2 is the concept of sequence tracks. A track can store a nucleotide or amino acid sequence, or a sequence annotation, such as the presence of a possible transcription factor binding site at a position. A sequence contains one or more tracks, each of which can provide an independent contribution to the scoring function. Figure 1 shows how an MSA can be constructed that includes multiple types of sequence annotations: one track contains the amino acid sequence, one track the secondary structure and one the matches against a specific motif pattern.

The track system of PRALINE 2 allows customization of the way in which sequences are scored, yet sometimes even more fine-grained control of the alignment algorithm itself is required. To this end PRALINE 2 was designed around a component architecture. Components can be thought of as modular building blocks of an MSA program; they receive inputs, perform some computation and return outputs, but cannot interact other than through these channels. The PRALINE 2 toolkit provides components for PSI-BLAST searches, a sequence regular expression matcher, and several non-standard alignment steps. The integration of existing tools, such as sequence-level prediction programs, is therefore straightforward.

## 3 Use cases

The new PRALINE 2 toolkit comprises a multiple sequence alignment strategy for protein sequences containing motifs, named Motif-Aware PRALINE (MA-PRALINE) (Dijkstra *et al.*, 2018). Using the multi-track scoring of PRALINE 2, a score boost is applied to traditional substitution scores when two symbols are part of a motif. MA-PRALINE can scan sequences for motifs in PROSITE pattern syntax (Hulo, 2006), or they can be provided manually. Motif annotations can be written in Jalview annotation format for visualization. MA-PRALINE was benchmarked against BAliBASE (Thompson *et al.*, 2005) families containing motifs; it was shown that alignment of motif regions is generally improved dramatically, while not significantly degrading the overall alignment structure. The motif annotation engine of PRALINE 2 was used on the HOMSTRAD reference benchmark set (Stebbings, 2004), to estimate the conservation signal encoded by motifs of varying lengths.

For nucleotide sequences, an early version of PRALINE 2 was used in the ConBind (Lelieveld *et al.*, 2016) transcription factor binding site (TFBS) conservation detection server. The ConBind server aligns homologous genomic regions from multiple species, with improved scoring of TFBS motifs. The output of ConBind shows which positions are conserved across species, and therefore which candidate TFBSs are most promising for further investigation. ConBind was validated by measuring gene activity with a luciferase reporter after knocking out candidate TFBSs, which were collected from a ChIPseq dataset. It was found that the higher motif alignment quality allowed ConBind to detect previously unknown cases of significant conservation.

The engine powering ConBind and MA-PRALINE is now available in the form of PRALINE 2, making the approach generically applicable. To facilitate the adoption of PRALINE, we have implemented a number of example scripts in the Github repository, showcasing how to apply it to common problems, including multi-track alignments, as well as more elaborate customizations.

*Conflict of Interest*: none declared.
